# Patient satisfaction with primary care physician performance in a multicultural population

**DOI:** 10.1186/s13584-020-00372-7

**Published:** 2020-03-25

**Authors:** Samah Hayek, Shany Derhy, Mathew Lee Smith, Samuel D. Towne, Shira Zelber-Sagi

**Affiliations:** 1 School of Public Health, University of Haifa, Haifa, Israel; 2 Memphis, USA; 3 Center for Population Health and Aging, Texas A&M University, College Station, TX 77843 USA; 4 Department of Environmental and Occupational Health, School of Public Health, Texas A &M University, College Station, TX 77843 USA; 5 Department of Health Promotion and Behavior, College of Public Health, The University of Georgia, Athens, GA 30602 USA; 6 Department of Health Management and Informatics, University of Central Florida, Orlando, FL 32816 USA; 7 Disability, Aging and Technology Faculty Cluster Initiative, University of Central Florida, Orlando, FL 32816 USA

**Keywords:** Primary care physicians’ performance, Ethnic differences, Patient satisfaction, Evaluation

## Abstract

**Background:**

A key component of the quality of health care is patient satisfaction, particularly in regard to Primary Care Physician (PCP), which represents the first contact with health care services. Patient satisfaction is associated with ethnic, regional and socio-demographic differences, due to differences in service quality, patient-doctor communication, and the patient’s perceptions. The aim of this study was to evaluate patients’ satisfaction related to primary care physicians’ (PCP) performance and to explore potential differences by ethnicity in a multicultural population.

**Methods:**

A national cross-sectional telephone survey was conducted, among a random sample of the Israeli population aged ≥25 years. Satisfaction level from performance of PCP was assessed using a validated questionnaire (30 items; 6 different domains).

**Results:**

The final sample included (*n* = 827 Jews; *n* = 605 Arabs, mean age 54.7(±14.9). In the adjusted logistic regression models, Arabs reported lower general satisfaction related to PCPs’ performance as compared to Jews (adjusted odds ratio (AOR), 0.63; (95% CI: 0.40–0.98). Arabs reported lower satisfaction related to PCPs’ performance across the following domains: communication skills (AOR, 0.42; 95% CI, 0.22–0.82); interpersonal manners (AOR, 0.37; 95% CI, 0.24–0.58); and time spent with the patients (AOR, 0.60; 95% CI, 0.43–0.85).

**Conclusions:**

Jews and Arabs were very satisfied with PCPs’ performance. However, there are ethnic differences in the extent of satisfaction level related to the performance of PCP. Satisfaction from PCPs’ performance may be achieved by improving the communication skills of the PCP, encouraging interpersonal interaction between the PCP and the patient, and devoting more time to the patient during the visits.

## Introduction

Patient satisfaction has emerged as an important measure in the evaluation of healthcare systems and in predicting health outcomes [1]. Healthcare systems are comprised of several complex and interrelated elements spanning multiple settings in which diverse patients seek care. This holds true in Israel, where Jews and Arabs make up the vast majority of residents. One of the many critical settings within the healthcare system is primary care where patients’ interactions may carry differences in perceived quality of healthcare or satisfaction [2, 3]. For example, an Israeli study found that Arabs reported a higher number of visits to primary care physician (PCP) compared to Jews, whereas the Jews utilize more specialists than the Arabs [4]. Further, Arabs have poorer health status compared to Jews, with higher rates of chronic diseases such as obesity [5], Diabetes [6], and poorer life expectancy [6]. Previous studies have examined the role of primary care in patient health and identified the high importance of the PCPs’ performance on health outcomes [3, 7], as well as on improved patient’s satisfaction [8]. Shi and colleagues found that primary care and income inequality were strong contributors to life expectancy [9]. Furthermore, patients who visited their PCP more often, and used them as the main source of information related to their health status, were more likely to be healthier (regardless of their initial health status and socio-demographic characteristics), hospitalized less, and spent less on annual healthcare expenditures [7]. In general, Israeli citizens report high satisfaction from the healthcare system; however, since 2009, the overall satisfaction level has not improved according to national surveys (across different ethnicities) [10, 11] In Israel, research in health care management and PCP performance field lacks evaluation of ethnic differences. Previous international studies have shown that racial and ethnic differences contribute to patients’ satisfaction levels with their PCP [12–15]. For example, Asian-American patients rate primary care performance lower than do white, African-Americans, and Hispanic [13]. Further, a study by Gross and Colleagues found that non-white patients were less satisfied with time spent with their physicians compared to white patients [16]. Racial and ethnic minorities in the US rate the quality of interpersonal care with physicians and the healthcare system lower relative to their non-Hispanic white counterparts [17–19].

Therefore, the purpose of this study was to explore whether patients’ satisfaction level regarding their PCPs’ performance differs across the two main population groups in Israel. The study may highlight performance domains that need to be improved in each population group in order to help meet the ethnicity-specific needs, which will hopefully lead to improved health outcomes of sub-populations in Israel.

## Methods

### Sampling strategy

A cross-sectional survey was employed by stratified random sampling of the adult Jews and Arabs population aged 25–75 years of age living in Israel. The Sample was done separately for Arabs and Jews. The sample from each population (Arabs and Jews) was drawn based on the proportion of the population of cities/towns/villages (rural and urban places) in Israel. We obtained the population size of each city or town from the Israeli Central Bureau of Statistics (CBS) list [20]. In Israel, city/town is the smallest district, which can be compared to the districts provided by the Israeli CBS. Inclusion criteria were participants aged ≥25 years old that were not currently employed by the Israeli defense forces. Individuals were excluded from the study if they did not consent to participate or were unable to complete a telephone interview (e.g., who may not have had the ability to complete the interview solely over the phone due to hearing impairment or some other issue).

The survey was conducted using telephone interviews in Hebrew and Arabic. Oral informed consent was obtained from each participant by telephone after a brief description of the study and questionnaire. Each interview lasted for approximately 15 min. Quality control procedures were implemented at all levels of data collection (training of staff, re-interviewing a sub-sample), data entry, and data analysis. The data about interviewees who could not be located were recorded to calculate the gross response rate. If the person answered the phone but refused to be interviewed, attempts were made to obtain basic demographic data from that person so comparisons between responders and non-responders could be made. For the current study, we contacted 2291 households, and 1867 were eligible to participate, 78 were not eligible, and outright 346 refused to talk. Among those who were eligible to participate, 1432 had full interviews, 114 partial interviews, 79 had difficulty in understanding the questions, and 242 were terminated after multiple postponements. The overall response rate for the current study was 62.5%. (Please see Supplementary Figure 1).

The justification of the sample size is as follows: For the prevalence estimates, with a precision of 95% Confidence Intervals of + 4%, allowing for an expected percentage of 43% of the sample reporting high satisfaction from medical competence [10], yields sample sizes of at least 590 subjects in each group. To validate the representativeness of the sampling strategy, we compared between study participants and the Israeli general population (Please see Supplementary Table 1).

### Survey instrument

A valid and reliable questionnaire, the Patient Satisfaction Questionnaire (PSQ III) [20, 21], served as the basis for our study. This survey was translated and amended to be culturally appropriate for the target population of Israel by the study team. A pre-test was conducted to assess the reliability and validity of the amended questionnaire. The internal consistency (reliability) of the questionnaire was high (Cronbach’s alpha = 0.87). The questionnaire included demographic characteristics and 32 items for estimating the satisfaction from the PCP performance. The performance measures of the PCP were calculated across each of the 6 domains: 1) general satisfaction; 2) technical skills; 3) accessibility and convenience; 4) communication; 5) interpersonal aspects; 6) and time spent with patients. Each statement for the performance measures was measured using a Likert-type scale (5 = strongly agree, 4 = agree, 3 = uncertain, 2 = disagree, 1 = strongly disagree). (Please see Supplementary Tables 3–4).

To estimate the level of satisfaction, we created an average score for each domain. According to the nature of variable and the study aims, we took the average of the answers per domain and categorized it into 5 groups based on the average range: 0–1 (1 = not satisfied at all), 1.01–2.0 (2 = not satisfied), 2.01–3 (3 = satisfied on average), 3.01–4.0 (4 = satisfied), 4.01–5.0 (5 = very satisfied).

#### Primary care physician performance measure domains

The current study focuses on six domains that are related to primary care physician performance measures from the patients’ point of view, and the overall satisfaction related to PCP performance. The internal consistency for each domain was reported in Supplementary Table 5.

##### General satisfaction

This scale consists of 3 items; including general satisfaction from the medical care patients receive from the PCP, and whether the patient would recommend his/her physician to a friend or family member.

##### Technical skills

This scale consists of 12 items; including questions about the PCP diagnosis skills, recommendations related to vaccines and promoting a healthy lifestyle.

##### Accessibility and convenience

This scale consists of 5 items; including waiting time at the doctor’s office, feasibility in terms of admitting to medical care in short notice without troubles, and conveniently accessible locations.

##### Communication

This scale consists of 5 items; including questions related to explaining and exemplifying everything to the patient, discussing the patient medical case.

##### Interpersonal aspects

This scale consists of 3 items; including questions related to the relationship between PCPs and patients.

##### Time spent with the patient

This scale consists of 2 items; including questions related to the time spent with patients.

### Statistical analyses

Descriptive statistics were conducted to describe participant characteristics by socio-demographics (e.g. age, gender, marital status, employment, and education), place of residence, and other relevant descriptive variables. To compare ethnic sub-groups, bivariate analyses were employed using t-tests to compare continuous variables and chi-square tests to compare categorical variables. The normal approximation was used to provide relevant confidence intervals (CI). To assess whether population group (Jews or Arabs, coded 0 or 1, respectively) was associated with patient satisfaction levels, we used multinomial logistic regression. Odds Ratios (OR) with 95% CIs were presented. Models were adjusted for age, BMI, education, HMO, smoking, having a permanent PCP, place of residence, and self-reported health status. In all domains, we had to merge responses for the “Not satisfied at all” category with the “Not satisfied” category, given small cell sizes to have a stable estimate with a reliable confidence interval. “On average satisfaction” (score of 2–3) was the reference group. We compared the extreme scores of the satisfaction to the average score, especially when a high number of participants reported that they were on average satisfied.

All statistical analyses were conducted using SAS (version 9.3. Cary, NC: SAS Institute Inc.; 2011). Two-sided *P*-values < 0.05 were considered statistically significant.

## Results

### Characteristics of the survey sample and comparison between Arabs and Jews participants

Of the 1423 participants, 42.5% were Arabs and 57.5% were Jews. Compared to Jews participants, Arabs were significantly younger [mean age 50.5 ± (SD = 13.4) vs. 57.8 ± (SD = 14.9)] and had significantly larger proportions of: lower education, being married or living with a partner, having children younger than age 18, and residence in the Northern district (Table 1).

**Table 1:** Demographic characteristics of the study population

		Jews (*N* = 827)	Arabs (*N* = 605)	*P-*value
Age mean (SD)		57.8 (14.9)	50.5 (13.4)	< 0.001
Age categorical	25–44	179 (21.9)	206 (34.2)	< 0.001
	45–64	335 (41.0)	302 (50.2)	
	65+	304 (37.1)	94 (15.6)	
Gender	Males	374 (45.2)	258 (42.6)	0.333
	Females	453 (54.8)	347 (57.4)	
Marital status	Unmarried	183 (22.2)	91 (15.0)	0.001
	Married/live with a partner	643 (77.8)	514 (85.0)	
Children under age 18		1.0 (1.6)	1.3 (1.7)	< 0.001
Employment	Employed	482 (58.6)	311 (51.5)	< 0.001
	Unemployed	22 (2.7)	46 (7.6)	
	Other (pensioner housewife, other)	319 (38.8)	247 (40.9)	
Education	Less than high school	280 (34.1)	316 (52.2)	< 0.001
	High school	109 (13.3)	109 (18.0)	
	Higher education	432 (52.6)	180 (29.8)	
Place of residence	Center	383 (46.4)	133 (22.0)	< 0.001
	North, South, Jerusalem (periphery)	442 (53.6)	471 (78.0)	

When comparing the utilization of health care system between Jews and Arabs, a significantly larger proportion of Arabs-belonged to Clalit HMO. Conversely, a significantly smaller proportion of Arabs purchased SHI and reported doctor visits during the last 6 months. Compared to their Jews counterparts, larger proportions of Arab participants reported higher rates of smoking, higher BMI, but perceived themselves to have better health status (Table 2). The Arab participants reported a higher proportion of receiving lifestyle advice during the past 12 months compared to Jews participants; 40.3% of the Arab participants reported receiving advice for weight loss or weight control compared to 28.2% Jews participants. About 47.9% of Arab participants received advice to increase physical activities compared to 43.9% Jews participants. Arab participants received more advice to reduce sodium or salt intake and reduce the amount of or calories in their diet compared to Jews participants (Supplementary Table 2).

**Table 2:** Utility of health care system and reported health status [n (%)]

		Jews (*N* = 827)	Arabs (*N* = 605)	*P*-value
HMO belonging	Clalit	388 (47.1)	448 (74.1)	< 0.001
	Maccabi	246 (29.8)	72 (11.9)	
	Meuhedet	118 (14.3)	48 (7.9)	
	Leumit	73 (8.8)	37 (6.1)	
Supplementary Health Insurance	Yes	730 (90.0)	386 (64.9)	< 0.001
	No	81 (10.0)	209 (35.1)	
Permanent primary care physician	Yes	782 (95.2)	587 (97.7)	0.023
	No	39 (4.8)	14 (2.3)	
Last visit to primary care	During the last 6 months	736 (89.8)	537 (89.6)	0.216
	6–12 months	42 (5.1)	22 (3.7)	
	More than 12 months	42 (5.1)	40 (6.7)	
Health status				
Smoking status	Current smoker	125 (15.1)	138 (22.8)	< 0.001
	Former smoker	32 (3.9)	34 (5.6)	
	No	669 (81.0)	433 (71.6)	
Having chronic disease	Yes	314 (38.6)	228 (38.1)	0.868
	No	513 (61.4)	377 (61.9)	
BMI mean (SD)		25.8 (±4.1)	27.2 (±4.5)	< 0.001
BMI category	< 25	346 (47.2)	197 (34.9)	< 0.001
	25–30	276 (37.7)	230 (40.7)	
	30+	111 (15.1)	138 (24.4)	
Perceived health status	Excellent / Very good	354 (43.6)	309 (51.9)	0.001
	Good	336 (41.3)	227 (38.2)	
	Fair / Poor	123 (15.1)	59 (9.9)	

### Satisfaction level of primary care physicians’ performance

Jews participants reported a higher proportion of “very satisfied” compared to Arabs regarding the performance of their PCP. Differences in satisfaction levels between Jews and Arabs were found across 5 out of 6 domains of PCPs’ performance domains: general satisfaction, communication skills, technical skills, interpersonal manners, and time spent with the doctor. The accessibility and convenience domain was the only domain where no significant difference was observed (Table 3).

**Table 3:** Satisfaction level from primary care physicians’ performance [n (%)]

		Jews (*n* = 827)	Arabs (*N* = 605)	*P*-value
General satisfaction^a^(Mean, SD)		4.24 (0.92)	4.13 (0.95)	0.037
General satisfaction^b^	Very satisfied^a^	509 (63.6)	335 (56.5)	0.013
	Satisfied	178 (22.2)	160 (27.0)	
	Satisfied on average level	81 (10.1)	73 (12.3)	
	Not satisfied	33 (4.1)	25 (4.2)	
Communication skills ^a^(Mean, SD)		4.51 (0.61)	4.37 (0.72)	< 0.001
Communication skills^b^	Very satisfied^a^	637 (79.6)	418 (70.5)	< 0.001
	Satisfied	134 (16.7)	134 (22.6)	
	Satisfied on average level	29 (3.6)	37 (6.2)	
	Not satisfied	1 (0.1)	4 (0.7)	
Technical skills^a^(Mean, SD)		3.85 (0.68)	3.76 (0.66)	0.019
Technical skills^b^	Very satisfied^a^	345 (43.1)	221 (37.3)	0.056
	Satisfied	347 (43.3)	286 (48.2)	
	Satisfied on average level	101 (12.6)	82 (13.8)	
	Not satisfied	8 (1.0)	4 (0.7)	
Interpersonal manners^a^(Mean, SD)		4.19 (0.80)	3.77 (0.76)	< 0.001
Interpersonal manners^b^	Very satisfied^a^	445 (55.6)	166 (28.0)	< 0.001
	Satisfied	268 (33.5)	323 (54.6)	
	Satisfied on average level	76 (9.5)	92 (15.5)	
	Not satisfied	11 (1.4)	11 (1.9)	
Time spent with doctor^a^(Mean, SD)		4.07 (0.04)	3.90 (0.04)	0.003
Time spent with doctor^b^	Very satisfied	428 (53.5)	259 (43.8)	< 0.001
	Satisfied	191 (23.9)	152 (25.7)	
	Satisfied on average level	134 (16.8)	147 (24.8)	
	Not satisfied	47 (5.9)	34 (5.7)	
Accessibility and convenience^a^(Mean, SD)		3.80 (0.76)	3.88 (0.75)	0.052
Accessibility and convenience^b^	Very satisfied	306 (38.2)	253 (42.7)	0.132
	Satisfied	352 (44.0)	239 (40.3)	
	Satisfied on average level	126 (15.7)	95 (16.0)	
	Not satisfied	17 (2.1)	6 (1.0)	

Notes^a^:The analysis was conducted on the continuous variables.^b^The analysis was conducted on the categorical variable. The range of the categories of satisfaction level: Not satisfied (range: 0–2.00), on average satisfied (range: 2.01–3.00), satisfied (range: 3.01–4.00), very satisfied (range: 4.01–5.00)

In models adjusted for age, BMI, education, HMO, smoking, having a permanent PCP, place of residence, and self-reported health status, Arabs compared to Jews were less likely to be “very satisfied” than “satisfied on average” with the general performance of their PCP (OR: 0.63;95% CI:0.40–0.98; *P*-value = 0.040), with the primary care physicians’ communication skills (OR:0.34;95% CI: 0.17–0.66; *P*-value = 0.001), with primary care physicians’ interpersonal manners (OR:0.32; 95% CI: 0.21–0.51; *P*-value< 0.001), and with time spent with the PCP (OR:0.56; 95% CI: 0.39–0.79; *P*-value = 0.001) (Table 4).

**Table 4:** Multivariate ordinal logistic regression^a^ for the association between ethnicity and satisfaction from primary care physicians and health care system in Israel

	AOR (95% CI)a Arabs/Jews	*P-*value
General satisfaction^b^		
Very satisfied	0.63 (0.40–0.98)	**0.040**
Satisfied	0.82 (0.48–1.39)	0.467
Satisfied on average level	**Ref**	
Not satisfied	0.67 (0.31–1.50)	0.338
Technical skills^b^		
Very satisfied	0.97 (0.64–1.48)	0.892
Satisfied	1.10 (0.73–1.66)	0.640
Satisfied on average level	**Ref**	
Not satisfied	1.28 (0.17–9.77)	0.808
Communication skills^b^		
Very satisfied	0.34 (0.17–0.66)	**0.001**
Satisfied	0.50 (0.25–1.02)	0.059
Satisfied on average level	**Ref**	
Not satisfied	2.12 (0.14–31.3)	0.605
Interpersonal manners^b^		
Very satisfied	0.32 (0.21–0.51)	**< 0.001**
Satisfied	1.00 (0.64–1.55)	0.998
Satisfied on average level	**Ref**	
Not satisfied	0.76 (0.23–2.48)	0.647
Time spent with doctor^b^		
Very satisfied	0.56 (0.39–0.79)	**0.001**
Satisfied	0.71 (0.47–1.05)	0.089
Satisfied on average level	**Ref**	
Not satisfied	0.81 (0.43–1.52)	0.508
Accessibility and convenience^b^		
Very satisfied	1.27 (0.85–1.88)	0.244
Satisfied	0.87 (0.59–1.29)	0.497
Satisfied on average level	**Ref**	
Not satisfied	0.39 (0.13–1.21)	0.101

Notes^a^:AOR (95%CI): Adjusted Odds ratio, 95%Confidence Interval. The model is adjusted for: age (years), BMI (kg/m2), education (less than high school, high school, and higher education), HMO, smoking status (current smoker, former smoker or no), having a permanent doctor, place of residence self-reported health status.^b^The range of the categories of satisfaction level: Not satisfied (range: 0–2.00), on average satisfied (range: 2.01–3.00), satisfied (range: 3.01–4.00), very satisfied (range: 4.01–5.00)

## Discussion

This study is unique in examining perceptions of satisfaction with PCP across ethnic subgroups within a country with national health insurance. Given all citizens have access to healthcare based on the socialized structure of the system, and all the HMOs are required to provide all members with the full range of benefits prescribed by the National Health Insurance law, and assuming that there are no ethnic differences in training and knowledge of the PCP in Israel, these ethnicity-based perceptions might explain possible disparities based on the quality of care received and practical availability of the healthcare services (e.g. in different geographic regions). Overall, Arabs compared to Jews were less likely to be “very satisfied” than “satisfied on average” with their PCPs’ performance across multiple domains (i.e. communication skills, interpersonal manners, and time spent with doctor). However, overall and across all the domains, most of the participants (Jews and Arabs) reported being either “satisfied” or “very satisfied” regarding the performance of their PCP. These observed differences by ethnic groups have been described in previous literature [22–25]. In our study, Arabs were younger, less educated and less employed compared to Jews participants. Previous studies have shown that personal characteristics may explain part of the ethnic differences in satisfaction from PCP, include age (younger ages report lower satisfaction), employment, income, and education [26]. Furthermore, previous data have shown that disparities in mental and physical health between minority groups in Israel could be explained, to a certain extent, by subjective and objective measures of SES [27]. Although, we adjusted for these variables in our multivariate analysis, there is still a possible residual confounding. Our results have shown that Arab participants reported a better-perceived health status than Jews. These results are consistent with previous data by Baron-Epel and colleagues, showing that Arabs tend to evaluate health better than Jews even though life expectancy is lower and morbidity is higher, concluding that subjective health status in Jews and Arabs does not necessarily have the same meaning in relation to objective measures of health [28]. We also assume that lower satisfaction from PCP performance can be due to differences between Arabs and Jews in the expectations from services received, but further studies are needed to explore disparities related to the expectations from PCP services and performance. For example, Arabs were less satisfied compared to Jews with the time spent with their PCP. This is a concern rising also from the PCP side. A previous study by Rosen and colleagues have shown that PCPs complain about patient visits being too short (averaging< 10 min), not having enough time to address mental and health promotion issues, the computer barriers between physician and the patient, and growing managerial monitoring/interference in their practice [29]. Interestingly, Arabs have reported receiving health promotion advice (e.g. physical activity, diet) from their PCP more commonly than Jews, which may be time-consuming and can explain the feeling of lack of sufficient visit time among Arab participants compared to Jews. However, there is no objective evidence to confirm that Arab patients receive less time compared to Jews patients and this should be explored in future studies.

Although not examined in this study, satisfaction may have been influenced by factors including physician-patient concordance (e.g., age, sex, ethnicity, and preferred language), the complexity of the patient’s health status and the perceived urgency of the patients’ health condition(s). The satisfaction level is often influenced by frustrations with the healthcare system and also reflect the disease profile or health status and sociodemographic characteristics of the patient [30], and ability to manage their conditions [31]. Disparities in patients’ satisfaction is an important component for measuring health care services and is associated with clinical outcomes, patient’s retention and treatment adherence. For example, a previous study among ambulatory patients has shown that the quality of interpersonal skills affects patient outcomes, and the patient adherence to treatment is mediated by patient satisfaction from their physicians [32]. Non-Adherence to treatment is a major public health concern and is associated with disease deterioration, poor prognosis, increase health care costs, and death [33]. This might further worsen the disparities in health status between Jews and Arabs, where Arabs have higher rates of obesity [5, 34], diabetes, [35, 36] sedentary behavior [37], smoking [38], and shorter life expectancy [6].

The current study has several strengths, such as having a large sample size and using a random selection from a large population. However, this study is also subject to several limitations. First, the use of random digit dialing restricted the results of the surveys to those with landlines, which may have resulted in selection bias. This does not seem to be a factor that would substantially affect the generalizability of the findings for the target population, supported also by the fact that our results are consistent with previous studies [11, 27, 28, 39]. Also, this study is subject to selection bias is given people that tend to answer telephone surveys may be different than people who refuse to answer telephone surveys. Further, our study is misrepresentative to the general Israeli populations in terms of age, over-representing people aged 65–74 years old, and under-presenting people aged 25–44 years old (Please see Supplementary Table 1). Recall bias was another limitation, given the use of self-reported surveys. However, this is a common practice in large national studies in other countries such as the US (e.g., Behavioral Risk Factor Surveillance System or BRFSS). Lastly, the health status of the participants, social support at home and health literacy were not collected and may be confounding factors.

The findings in the current study also hold insights for global stakeholders interested in understanding health disparities across socio-demographics, namely ethnicity. Key theoretical frameworks (e.g., the World Health Organization’s Framework for Action on the Social Determinants of Health) [40], have identified race and ethnicity, among other factors such as public policy (e.g., health policy), socioeconomic position, and education as critical in identifying health inequities. In terms of racial or ethnic disparities in patient satisfaction, early reports from the US such as the Institute of Medicine’s report, *Unequal Treatment*, highlighted several gaps in the quality of care received across race and ethnicity even with similar health care coverage [41]. Our results are also consistent with other studies in that ethnic minority groups rate the interpersonal care by physicians (and within the healthcare system) lower compared to their counterparts within majority sub-groups [18, 19]. For example, our finding that Arabs reported lower satisfaction with their PCPs’ interpersonal manners compared to Jews mirrors findings from Johnson and colleagues examining patient-physician communication differences between African-American and non-Hispanic white patients [17]. While this study provides a glimpse into healthcare satisfaction and possible underlying inequities among patient sub-groups in Israel, findings point to the need for future studies and specific recommendations for improving patient-provider interactions.

## Conclusions

In summary, for both Jews and Arabs, the satisfaction level from the PCPs’ performance is above the average. However, the current study found that there are significant differences between Jews and Arabs in the extent of satisfaction level from the PCPs’ performance and from the healthcare system; Jews are more very satisfied with their PCPs’ performance compared to Arabs. Our findings show that ethnic differences are evident with the communication domains, interpersonal manners, and time spent with the doctor. These differences might contribute to ethnic differences in health outcomes.

### Policy implications and recommendations

This study has implications for national and international policy health leaders. In some countries with national health insurance, like Israel, PCPs are the gatekeepers of the healthcare system. For this reason, differences in the satisfaction level from PCP warrant concern as potential contributors to disparities in clinical outcomes, patient retention and adherence to treatment, which leads to disparities in life expectancy. There is a need for further research aimed for a comprehensive understanding of the multiple factors that underlie these differences in satisfaction and its potential clinical implications. It is important to note that patient satisfaction from PCP’s performance is based on the patient’s perception of healthcare services and the interaction between patient and PCP. Thus, new studies should focus on exploring the reasons for these differences in perceptions and should take into account both patient satisfaction from PCP’s performance and objective performance measures that the healthcare system defines.

In the meantime, efforts should be focused on PCP-patient interactions including interventions to increase patient’s health literacy, improve PCPs’ interpersonal skills (e.g. listening, empathy toward patients, emotional support, and friendliness), increase the time spent with the patients, and training professionals to be culturally competent and understand their patients’ needs.

## Supplementary information

**Additional file 1.**

**Additional file 2.**

**Additional file 3.**

## Data Availability

Data will be provided upon request.

## Abbreviations

PCP: Primary Care Physician
PCPs’ performance: Primary Care Physicians’ performance
PSQ: Patient Satisfaction Questionnaire
HMO: Health Maintenance Organization
