# Supplementary figures and images for: Patient satisfaction with primary care physician performance in a multicultural population
Source: Isr J Health Policy Res. 2020 Mar 25;9:13. doi: 10.1186/s13584-020-00372-7 (PMC7098152; doi:10.1186/s13584-020-00372-7)

## Slide 1
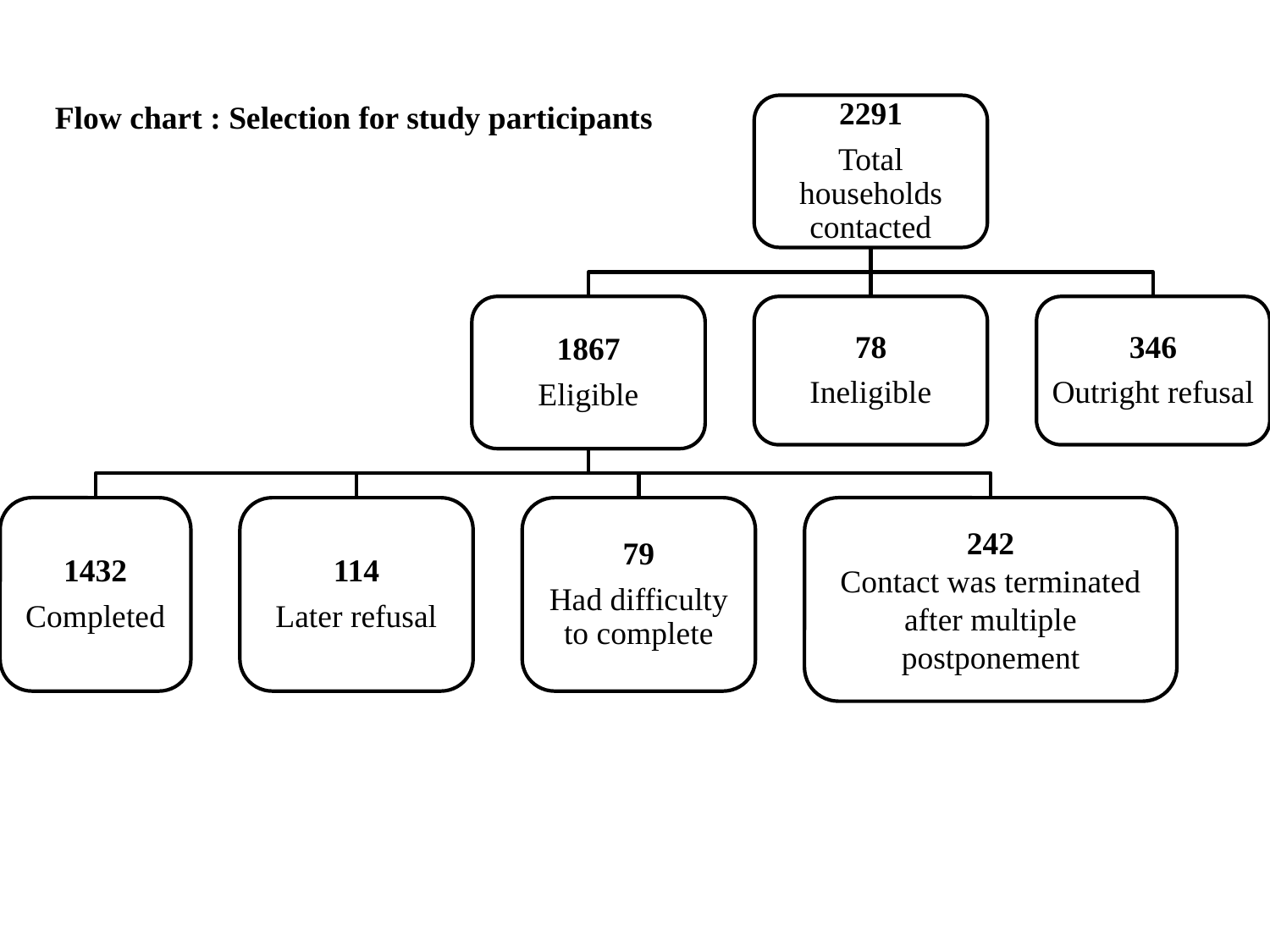

Flow chart : Selection for study participants

Supplement: Supplementary file 1 — Additional file 1. [file 13584_2020_372_MOESM1_ESM.pptx]
