# Supplementary material for: Patient satisfaction with primary care physician performance in a multicultural population
Source: Isr J Health Policy Res. 2020 Mar 25;9:13. doi: 10.1186/s13584-020-00372-7 (PMC7098152; doi:10.1186/s13584-020-00372-7)
Supplement: Supplementary file 2 — Additional file 2. [file 13584_2020_372_MOESM2_ESM.docx]

**Supplementary table1: Demographic characteristics of the Israeli population compared to the study population**

|  |  | Israeli population  (N=4,472,700) | Study population  (N=1432) |
| --- | --- | --- | --- |
| Ethnicity¹ |  |  |  |
|  | Jewish (%) | 82.0 | 57.8 |
|  | Arabs (%) | 18.0 | 42.2 |
| Age¹ |  |  |  |
|  | 25-44 (%) | 51.1 | 27.1 |
|  | 45-64 (%) | 36.4 | 44.9 |
|  | 65-74 (%) | 12.4 | 28.0 |
| Place of residence² |  |  |  |
|  | Center (%) | 40.7 | 22.0 |
|  | North, South, Jerusalem (%) (periphery) | 59.3 | 78.0 |

Notes : Data related the Israeli population was abstracted from the Israeli Center Bureau of Statistics (ICBS) report: <https://www.cbs.gov.il/he/publications/doclib/2018/rep_10/part01_h.pdf>.

¹Data related to ethnicity and age was presented among population age 25-75 years old.

²Place of residence: Data is presented for all age-age groups. In our study we combined the center district and Tel-Aviv district as a one district, whereas the ICBS reports separate between them.

**Supplementary table2: Ethnic differences in patients reported physician’s lifestyle advice [n, %]**

|  |  | Jewish  (N=827) | Arabs  (N=605) | P |
| --- | --- | --- | --- | --- |
| To lower your risk for certain disease, during the past 12 months have you ever been told by a doctor or health professionals at the HMO to control your weight or lose weight? | | | |  |
|  | Yes | 229(28.2) | 241(40.3) | <0.001 |
|  | No | 552(68.0) | 336(56.2) |  |
|  | Irrelevant* | 31(3.8) | 21(3.5) |  |
| To lower your risk for certain disease, during the past 12 months have you ever been told by a doctor or health professionals at the HMO to increase your physical activity or exercise? | | | |  |
|  | Yes | 354(43.6) | 286(47.9) | 0.09 |
|  | No | 415(51.1) | 291(48.7) |  |
|  | Irrelevant | 43(5.3) | 20(3.4) |  |
| To lower your risk for certain disease, during the past 12 months have you ever been told by a doctor or health professionals at the HMO to reduce the amount of sodium or salt in your diet? | | | |  |
|  | Yes | 197(24.4) | 189(31.7) | <0.001 |
|  | No | 583(72.3) | 387(64.8) |  |
|  | Irrelevant | 26(3.2) | 21(3.5) |  |
| To lower your risk for certain disease, during the past 12 months have you ever been told by a doctor or health professionals at the HMO to reduce the amount of fat or calories in your diet? | | | |  |
|  | Yes | 248(30.7) | 288(48.4) | <0.001 |
|  | No | 535(66.3) | 294(49.4) |  |
|  | Irrelevant | 24(3.0) | 13(2.2) |  |

Notes: *Irrelevant: participants reported that the question is not relevant to them.
