# Supplementary material for: Patient satisfaction with primary care physician performance in a multicultural population
Source: Isr J Health Policy Res. 2020 Mar 25;9:13. doi: 10.1186/s13584-020-00372-7 (PMC7098152; doi:10.1186/s13584-020-00372-7)
Supplement: Supplementary file 3 — Additional file 3. [file 13584_2020_372_MOESM3_ESM.docx]

**Supplementary 3-Questionnaire**

**Questionnaire no.** ____________

Haifa University- School of Public Health

**Interviewer –after you are seated, and ready to begin, read:**

**“**Hello**,** my name is _______. Thank you for your willingness to answer some questions regarding to your relationship and satisfaction with your primary care physician. If, at any stage during the interview, any of the questions is unclear, please tell me. We will begin the interview with some questions about demography, habits, health status and finally about your evaluation and rating of your primary care physician.

**Interviewer Name: ____________ Interviewer Code _______**

**Telephone:** _____________________.

**Date of Interview: ________________** (Day/Month/Year)**.**

**Part I: Questions on socio-demographic characteristics**

1. **Date of birth according to Identification document**: |__|__||__|__||__|__| (Day/Month/Year)
2. **Age**: |__|__|__|.

1. **Gender**:
2. Male
3. Female
4. **Marital Status**:
5. Single
6. Currently married or with a life partner
7. Divorced and currently not remarried
8. Widowed and currently not remarried
9. **Number of children** **under 18**: |__|__|
10. **Country of birth**:
11. Israel
12. Other

**6.1 If other, then year of immigration**: |__|__|__| |__

1. **What has your occupational status been during the past month**:
2. Salaried employment
3. Self-employed
4. Unemployed with a source of income.
5. Unemployment with no external income.
6. Pensioner
7. Housewife.
8. Other __________________.

**7.1 If unemployed, did you work in the past**:

1. Yes, as an employee
2. Yes, as self-employed
3. I did not work in the past
4. Other, specify: _________________________

**8. In comparison with other people lives around you- How do you define your income?**

- 1. Above the average
  2. On the average
  3. Below the average
  4. Refused

5. Don’t know

**9. What is your highest degree?**

1. Less than high school

2. High school without Bagrut

3. High school with Bagrut

4. Professional degree

5. BA

6. Master degree and higher

7. Bible studies

8. Do not know

**10. Where do you live in Israel?**

- 1. North
  2. Center
  3. South
  4. Jerusalem

**10.1 Please – tell us the name of the city**: ________________________

**11. To which HMO (Kupat Holim) do you belong to?**

1. Clalit
2. Maccabi
3. Meuhedet
4. Leumit

**12. Do you have supplementary health insurance (SHI), through your health fund**?

1. Yes
2. No
3. Don’t know/ don’t remember

**13. Do you have any chronic disease such as diabetes, hypertension, or cholesterol?**

1. Yes

2. No

3. Do not Know / Refused to answer

**Part II:**

This section will discuss general information about your primary care doctor

**14.** **Do you have a permanent primary care physician?**

1. Yes (Go to question 16)

2. No (Go to section 3)

3. Do not know

**14.1** **If you don’t have a primary care physician – can you describe the reasons ___________________________**

**15. When was your last visit to your primary care physician?**

1. Last 30 days

2 1-3 months ago

3. 3-6 months ago

4. 6-12 months ago

5. 2 years ago

**Part III: Questions related to performance measure indicators**

(Please notice that all the questions are related to the primary care physician)

How strongly do you agree or disagree with each of the following statements?

| Strongly disagree | Disagree | Uncertain | Agree | Strongly Agree |  |  |
| --- | --- | --- | --- | --- | --- | --- |
|  |  |  |  |  | My doctor never exposes me to unnecessary risk | 1 |
|  |  |  |  |  | My primary care doctor needs to be more thorough in treating and examining me | 2 |
|  |  |  |  |  | I am very satisfied with the medical care I receive from my doctor | 3 |
|  |  |  |  |  | My doctor is good to explain the reason for medical tests | 4 |
|  |  |  |  |  | I am usually kept waiting for a long time when I am at the doctor ‘s office | 5 |
|  |  |  |  |  | I think my doctor’s office has everything needed to provide complete medical care | 6 |
|  |  |  |  |  | Sometimes my doctor make me wonder if his/her diagnosis is correct | 7 |
|  |  |  |  |  | During my medical visit, I am always allowed to say everything that I think is important | 8 |
|  |  |  |  |  | When I go to my doctor, my doctor is careful to check everything when treating and examining me | 9 |
|  |  |  |  |  | It is hard for me to get medical care on short notice | 10 |
|  |  |  |  |  | Sometimes my doctor use medical terms without explaining what they mean | 11 |
|  |  |  |  |  | I have easy access to the medical specialists I need | 12 |
|  |  |  |  |  | In the HMO (or clinic) that I get my medical care, people have to wait too long for **emergency** treatment | 13 |
|  |  |  |  |  | My doctor acts too businesslike and impersonal toward me | 14 |
|  |  |  |  |  | There is a crisis in health care system and in the HMOs in Israel today | 15 |
|  |  |  |  |  | If I need hospital care, I can get admitted without any troubles or delays | 16 |
|  |  |  |  |  | My doctor treat me in a very friendly and courteous manner | 17 |
|  |  |  |  |  | Sometimes my doctor hurry too much when he treats me | 18 |
|  |  |  |  |  | Some of the primary care doctors that I have seen lack experience with my medical problems | 19 |
|  |  |  |  |  | Places where I can get medical care are very conveniently located | 20 |
|  |  |  |  |  | The doctor sometime ignore what I tell him/her | 21 |
|  |  |  |  |  | My doctor rarely gives me advice about ways to avoid illness and stay healthy | 22 |
|  |  |  |  |  | (Ask this question just if the participant is a smoker)  My doctor talks with you about quitting smoking when I visit him/her | 23 |
|  |  |  |  |  | My doctor always recommends me to get the flu vaccine | 24 |
|  |  |  |  |  | My doctor listens carefully to what I have to say | 25 |
|  |  |  |  |  | My doctor usually spend plenty of time with me | 26 |
|  |  |  |  |  | My doctor always does his best to keep me from worrying | 27 |
|  |  |  |  |  | I am dissatisfied with some things about the medical care I receive | 28 |
|  |  |  |  |  | My doctor is very competent and well trained | 29 |
|  |  |  |  |  | I have an easy access to the medical specialists I need | 30 |
|  |  |  |  |  | I would recommend my doctor to all my friends and relatives | 31 |
|  |  |  |  |  | My doctor routinely recommends me to lose weight/ maintain a healthy weight and lifestyle | 32 |

**Supplementary table 4 : Patient satisfaction questionnaire item by aspect of satisfaction (Domain)**

| **Statement** | **Aspect of satisfaction** | **A** |
| --- | --- | --- |
| My doctor never exposes me to unnecessary risk | Technical Quality |  |
| My doctor needs to be more thorough in treating and examining me | Technical Quality |  |
| I am very satisfied with the medical care I receive from my doctor | General Satisfaction |  |
| My doctor is good in explaining the reasons for medical tests and exams | Communication |  |
| I am usually kept waiting for a long time when I am at the doctor ‘s office | Accessibility and Convenience |  |
| I think my doctor’s office is well equipped to provide complete medical care | Technical Quality |  |
| Sometimes my doctor makes me wonder if his/her diagnosis is correct | Technical Quality |  |
| During my medical visit, I am always allowed to say everything that I think is important | Communication |  |
| When I go to my doctor, my doctor is careful to check everything while treating and examining me | Technical Quality |  |
| It is hard for me to get my medical care on short notice | Accessibility and Convenience |  |
| Sometime my doctor uses medical terms without explaining what they mean | Communication |  |
| I have feasible access to the medical specialists I need | Accessibility and Convenience |  |
| In the HMO (or clinic) where I get my medical care, people have to wait too long for emergency care (e.g., high fever and/or feeling badly sick) | Accessibility and Convenience |  |
| My doctor acts too businesslike and impersonal toward me | Interpersonal Aspects |  |
| If I need a hospital care, I can get be admitted without any troubles or delays | Accessibility and Convenience |  |
| My doctor treats me in a very friendly and courteous manner | Interpersonal Aspects |  |
| Sometimes my doctor hurry too much when he treats me | Time Spent with Doctor |  |
| My doctor lacks experience with my medical problems | Technical Quality |  |
| Most of the places where I can get medical care are very conveniently located | Accessibility and Convenience |  |
| My doctor sometime ignores what I tell him/her | Communication |  |
| My doctor **rarely** gives me advice about ways to avoid illness and stay healthy | Technical Quality |  |
| [If the participant is a smoker]  My doctor talks with me about quitting smoking when I visit him/her | Technical Quality |  |
| My doctor always recommends me to get the seasonal flu vaccine | Technical Quality |  |
| My doctor listens carefully to what I say | Communication |  |
| My doctor usually spends plenty of time with me | Time Spent with Doctor |  |
| My doctor always does his best to keep me from worrying | Interpersonal Aspects |  |
| I am dissatisfied with some things about the medical care I receive | General Satisfaction |  |
| My doctor is very competent and well trained | Technical Quality |  |
| I would recommend my doctor to my friends and relatives | General Satisfaction |  |
| My doctor routinely recommends me to lose weight/ maintain a healthy weight | Technical Quality |  |
| My doctor routinely recommends me to maintain a healthy lifestyle | Technical Quality |  |

Supplementary table 5: Internal consistency for patient satisfaction domains

| Cronbach’s alpha | Patient satisfaction domain |
| --- | --- |
| 0.69 | General satisfaction |
| 0.68 | Technical skills |
| 0.58 | Accessibility and convenience |
| 0.66 | Communication |
| 0.40 | Interpersonal aspects |
| 0.44 | Time spent with the patients |
